# 

**Published:** 1891-08-15

**Authors:** 


PRICE ONE PENNY.

No. 255, Vol. X.—August 15th, 1891.

[Registered at the General Post Office as a Newspaper for transmission in the United Kingdom and Abroad.]

### THE HOSPITAL

WITH EXTRA NURSING SUPPLEMENT.

Per Annum, 6s. 6d.; post free.

Monthly Parts, 6d.; post free 8d.

Ditto per Annum, 8s., post free.

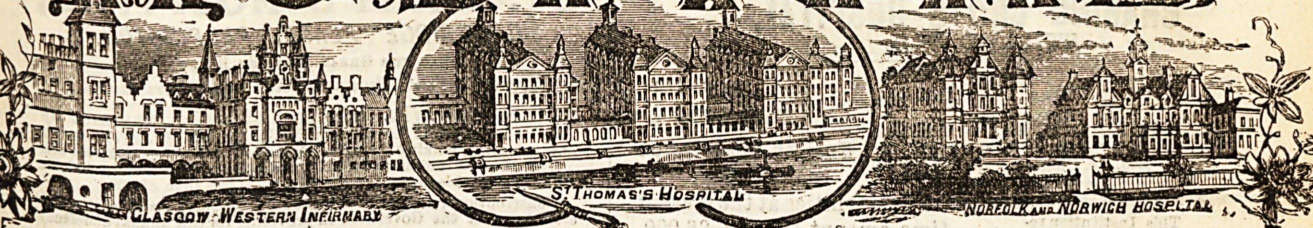

A WEEKLY JOURNAL OF
Science, Medicine, Nursing and Philanthropy.

### SATURDAY, AUGUST 15th, 1891.

**English Lunacy in 1890** . . . . . . . . 229

**"Mad Doctors"—A Protest.** By A. R. Urquhart, M.D. 230

**Passing Topics.**—The Personal Element . . . . 230

**Recreation in Relation to the Health of the People.** VII.—Among the Mining Population . . . . . . 231

**The Story of the Insane from Year to Year** . . 232

**The History and Capabilities of Herbal Simples.** By W. T. Fernie, M.D. XXXVI.—Ferns . . . . . 232

**Everybody's Page** . . . . . . . . . 233

**Notes and News** . . . . . . . . . 234

**General Charities** . . . . . . . . . 235

**Scraps and Gleanings** . . . . . . . . 235

**Annotations.** — Hospitals and Telephones — Holidays—Artizans' Cookery—Our Children's Ears . . . . 236

**The Practitioner's Mirror and Retrospect**—

Medicine.—Rheumatic Hyperpyrexia—Gastrectasis . . 237

Surgery.—Diseases of the Fingers: C—Contractions . [illegible]33

Otology.—Treatment of Otorrhœa . . . . . . 239

**The International Congress of Hygiene and Demography** . . . . . . . . . . 240

**The Student of Medicine.**—On German Students (*concluded*)—Notes from the Schools—Students' Medical Society—Appointments—Vacancies—Scholarships and Prizes—Pass Lists—Athletics.

### EXTRA SUPPLEMENT—THE NURSING MIRROR.

**En Passant.**—Sister Frances—Middlesbrough Home—Norwich Staff of Nurses—University Nurses—The Princess of Wales and the Nurses . . . . . . . . . cxiii

**The Story of Sister Frances** (*With Portraits*) . . cxiv

**For Reading to the Sick**—Contentment.—I. . . . cxv

**Nurses and their Ways** . . . . . . . . cxv

**American News** . . . . . . . . . cxvi

**Notes and Queries** . . . . . . . . . cxvi

**Everybody's Opinion.**—District Nursing . . . . cxvii

**Appointments** . . . . . . . . . . cxvii

**Christmas Competitions** . . . . . . . cxvii

**The Princess of Wales' Fund for Mrs. Grimwood** . cxvii

**Presentation** . . . . . . . . . . cxvii

**Amusements and Relaxation** . . . . . . cxvii

**Off Duty.**—A Bad Quarter of an Hour . . . . cxviii

**Death in Our Ranks** . . . . . . . . cxviii

